# Sensitivity analysis for random measurement error using regression calibration and simulation-extrapolation

**DOI:** 10.1016/j.gloepi.2021.100067

**Published:** 2021-11-21

**Authors:** Linda Nab, Rolf H.H. Groenwold

**Affiliations:** a Department of Clinical Epidemiology, Leiden University Medical Center, Leiden, the Netherlands; b Department of Biomedical Data Sciences, Leiden University Medical Center, Leiden, the Netherlands

**Keywords:** Classical measurement error, Sensitivity analysis, Quantitative bias analysis, Regression calibration, Simulation-extrapolation

## Abstract

**Objective:**

Sensitivity analysis for random measurement error can be applied in the absence of validation data by means of regression calibration and simulation-extrapolation. These have not been compared for this purpose.

**Study design and setting:**

A simulation study was conducted comparing the performance of regression calibration and simulation-extrapolation for linear and logistic regression. The performance of the two methods was evaluated in terms of bias, mean squared error (MSE) and confidence interval coverage, for various values of reliability of the error-prone measurement (0.05–0.91), sample size (125–4000), number of replicates (2−10), and R-squared (0.03–0.75). It was assumed that no validation data were available about the error-free measures, while correct information about the measurement error variance was available.

**Results:**

Regression calibration was unbiased while simulation-extrapolation was biased: median bias was 0.8% (interquartile range (IQR): −0.6;1.7%), and −19.0% (IQR: −46.4;−12.4%), respectively. A small gain in efficiency was observed for simulation-extrapolation (median MSE: 0.005, IQR: 0.004;0.006) versus regression calibration (median MSE: 0.006, IQR: 0.005;0.009). Confidence interval coverage was at the nominal level of 95% for regression calibration, and smaller than 95% for simulation-extrapolation (median coverage: 85%, IQR: 73;93%). The application of regression calibration and simulation-extrapolation for a sensitivity analysis was illustrated using an example of blood pressure and kidney function.

**Conclusion:**

Our results support the use of regression calibration over simulation-extrapolation for sensitivity analysis for random measurement error.

## Introduction

Measurement error is common in biomedical research but often ignored [1,2]. When ignored, measurement error can lead to considerable biases in exposure-outcome associations [3]. Random measurement error in the exposure variable, also known as 'classical’ measurement error, occurs when the measured exposure is distributed around the true exposure with independent error and, is common in various domains of epidemiology [[4], [5], [6]]. Random measurement error in an exposure variable introduces bias in the exposure-outcome association, which is sometimes referred to as attenuation bias [7] or regression dilution bias [8,9].

Various methods for measurement error correction are available [[10], [11], [12], [13], [14], [15], [16], [17], [18]], yet application of these methods is rare in biomedical research [1,2]. One possible barrier is the necessity of for instance validation data, which are often unavailable [5]. Validation data can be used to estimate the measurement error model and its parameters, and subsequently used for measurement error correction.

In the absence of validation data, regression calibration [19,20] and simulation-extrapolation [15], among other, can be applied to correct for random exposure measurement error. Both methods only require assumptions about the variance of the random measurement error, for example based on literature or expert knowledge. Regression calibration in the absence of validation data is available in the *R* [21] package *mecor* for measurement error correction [22] that implements the regression calibration described by Rosner et al. [23]. Alternatively, simulation-extrapolation is easy to use due to its implementation in the *R* package *simex* [24] and the *simex* procedure [25] in *Stata* [26].

Simulation-extrapolation and regression calibration have been compared in simulation studies for scenarios where replicate measures of the error-prone exposure were available [5,27,28]. The studies by Perrier et al. [5], Batistatou et al. [27] and Fung et al. [28] were consistent and showed that, regression calibration and simulation-extrapolation reduced bias compared to when no measurement error correction was applied or when the replicate exposure measures were pooled. It was also shown that application of simulation-extrapolation generally produced more biased effect estimates than regression calibration, especially when the reliability of the error-prone measure was low.

Perrier et al. [5] and Batistatou et al. [27] studied a univariable linear regression in a limited number of scenarios, e.g., large sample sizes, and limited range of reliability of the error-prone measure. Fung et al. [28] studied a multivariable logistic regression in a limited number of scenarios, e.g., not varying sample size and a limited range of reliability. Further investigation is needed of the performance of regression calibration and simulation-extrapolation in more complex settings, as typically found in epidemiologic research (e.g., multivariable linear and logistic regression, varying sample size and levels of reliability). Moreover, since the previous simulation studies focused on settings where replicate measures were available, we aim to research how their results translate to settings where no replicate measures, but only an estimate of the measurement error variance is available. The quantification of the performance of the two methods in this broader range of settings is used as the input for a framework guiding the application of sensitivity analysis for random measurement error.

This paper is structured as follows. Section 2 reviews and applies regression calibration and simulation-extrapolation by using two motivating examples of a linear regression and logistic regression where the exposure is prone to error. In Section 3, a simulation study is described that aims to compare regression calibration and simulation-extrapolation for linear regression and logistic regression, and results from the simulation study are shown. Section 4 introduces a framework for conducting sensitivity analysis, also known as quantitative bias analysis [29], for random measurement error by means of regression calibration and simulation-extrapolation. We conclude with a discussion of our results and recommendations in Section 5.

## Review and motivating example

When an exposure variable is measured with random measurement error, the exposure-outcome association is biased. In a univariable model with a continuous outcome, under the assumption of random measurement error, the uncorrected effect estimate is biased by a factor equal to the variance of the true measure divided by the sum of the variance of the true measure and the measurement error variance. This is sometimes referred to as the ‘attenuation factor’ because the variance of the true measure plus the measurement error variance is always greater than the variance of the true measure alone [7]. For a linear regression, this can be expanded to the multivariable case by conditioning on the covariates in the multivariable model. For a logistic regression, the bias induced by random measurement error cannot be quantified exactly [13]. Kuha [30] shows that under the assumption that the effect of the exposure on the outcome is ‘small to moderate’ and/or the measurement error is ‘small’, the uncorrected effect estimate in a logistic regression is biased approximately by the attenuation factor. We refer to Kuha for a detailed discussion.

### Review of regression calibration and simulation-extrapolation

In a linear regression or logistic regression, the random measurement error in an exposure can be corrected by application of regression calibration and simulation-extrapolation. Regression calibration starts by estimating the uncorrected effect of the error-prone measure on the outcome (in a multivariable model, given the covariates). Subsequently, the uncorrected estimate is multiplied by the inverse of the attenuation factor: the estimated variance of the error-prone exposure (given the covariates), divided by the estimated variance of the error-prone exposure (given the covariates) minus the measurement error variance. The measurement error variance can be estimated by using e.g., replicate measurements, by estimating the within individual variance and averaging over all individuals. Alternatively, the measurement error variance could be informed by e.g., external data or expert knowledge.

Simulation-extrapolation consists of two steps. In the simulation step, extra measurement error is added to the error-prone exposure. The size of this extra measurement error is usually 0.5, 1, 1.5 and 2 times the measurement error variance. Using these simulated exposure measurements with extra added measurement error, the outcome model is estimated. This is repeated 100 times for each value of the extra measurement error variance and the newly obtained estimates are averaged. Then, in the extrapolation step, a model (e.g., linear, quadratic) is fitted through the effect estimates for the varying sizes of the measurement error. The corrected effect estimate is then obtained by extrapolating the fitted model to the situation where the measurement error is equal to 0. For a visualisation of simulation-extrapolation, see e.g. Keogh et al. [3].

### Motivating example

Hereafter, regression calibration and simulation-extrapolation to correct for random measurement error are demonstrated for linear regression and logistic regression, using an example about the association between systolic blood pressure and kidney function (serum creatinine) and an example about the association between sodium intake and hypertension, respectively.

Example 1 Linear regression of blood pressure and kidney function in pregnant women

For the first example, we used data of retrospective records of all women who attended a tertiary maternity hospital pregnancy day assessment clinic over a 6-month period in 2014 in Australia [31]. Care always included serial, manual blood pressure measurements every 30 min by registered midwives using aneroid sphygmomanometers. Serum creatinine and demographic data were obtained using routinely collected data. One woman with a serum creatinine level lower than 10 μmol/L was excluded from the analysis.

First, the association between systolic blood pressure and serum creatinine was determined by only using the systolic blood pressure measurement obtained after 30 min. The association was adjusted for age. We found that an increase of 10 mmHg in systolic blood pressure was associated with a 1.18 μmol/L (95% CI: 0.14;2.23) increase in serum creatinine (Table 1). In this analysis, the random measurement error in the single systolic blood pressure measurement was not taken into account. Using the four consecutive blood pressure measurements (obtained after 30, 60, 90 and 120 min), it was found that the within individual variance of the systolic blood pressure measures was on average 48.3 mmHg, resulting in a reliability of 0.6. The within individual variance of 48.3 mmHg was subsequently used to correct for the random measurement error in the single systolic blood pressure measurement using regression calibration and simulation-extrapolation, while adjusting for age. Using regression calibration, we found that an increase of 10 mmHg was associated with a 2.04 μmol/L (95% CI: 0.22;4.23) increase in serum creatinine (Table 1). Using simulation-extrapolation, we found that an increase of 10 mmHg was associated with a 1.67 μmol/L (95% CI: 0.15;3.29) increase in serum creatinine (Table 1).

Example 2 Logistic regression of sodium intake and hypertension in adults

**Table 1:** Effect estimates (95% confidence intervals) of the association between blood pressure (systolic blood pressure, per 10 mmHg) and kidney function (serum creatinine, μmol/L) (linear regression, Example 1) and the association between sodium intake (per gram) and hypertension (odds ratio obtained from a logistic regression, Example 2). The uncorrected effect estimates are obtained by using the first measurement only, the corrected estimates are obtained by using the three consecutive blood pressure measurements (Example 1) and the second consecutive sodium intake measurement (Example 2).

Example	Uncorrected	Regression Calibration	Simulation-Extrapolation
Systolic blood pressure and kidney function	1.18^b^ (0.14;2.23)	2.04 (0.22;4.23)	1.67 (0.15;3.29)
Sodium intake and hypertension^a^	1.04^c^ (1.00;1.09)	1.12 (0.99;1.27)	1.07 (1.00;1.16)

Estimates were obtained from the pregnancy day and assessment clinic study [31] (systolic blood pressure and kidney function, reliability of the error-prone blood pressure measurement: 0.6) and the national health and nutrition examination survey [32] (sodium intake and hypertension, reliability of the error-prone sodium intake measurement: 0.4).

a Odds ratio.

b Estimate is corrected for age, but not for the measurement error in systolic blood pressure.

c Estimate is corrected for age and body mass index, but not for the measurement error in sodium intake.

For the second example, we used data of the 2015–2016 cycle of the National Health And Nutrition Examination Survey [32]. Given natural variation of sodium intake within individuals, a single measurement of sodium intake often does not reflect the true level of sodium intake. In the NHANES, two sodium intake measurements were taken using a 24-hour recall. The first dietary recall interview was collected in-person and the second interview was collected by telephone 3 to 10 days later. Participants' hypertension status was based on a combination of their self-reported history of any diagnosis of hypertension and self-reported use of prescribed hypertension medication. Demographic information was collected using the family and sample person demographics questionnaires in the home, by trained interviewers. Weight and height were measured by trained health professionals. For this analysis, participants between 18 and 80 years were included. Additionally, all participants with a body mass index (BMI) higher than 55 and a sodium intake of more than 10 g per day were excluded from the analysis.

First, the association between sodium intake and hypertension was determined by only using the first sodium intake measurement. The association was adjusted for BMI and age. It was found that an increase of 1 g in sodium intake was associated with a 1.04 (95% CI: 1.00;1.09) times increase in the odds for hypertension. In the NHANES data, the within individual variation of sodium intake was on average 1.7 g, which was obtained by using the two consecutive sodium intake measures, resulting in a reliability of 0.4. The within individual variance of 1.7 was subsequently used to correct for the random measurement error in the first sodium intake measure using regression calibration and simulation-extrapolation, while adjusting for age. Using regression calibration, we found that an increase of 1 g in sodium intake was associated with a 1.12 (95% CI: 0.99;1.27) increase in the odds for hypertension (Table 1). Using simulation-extrapolation, we found that an increase of 1 g in sodium intake was associated with a 1.07 (95% CI: 1.00;1.16) increase in the odds for hypertension (Table 1).

## Simulation study

To investigate the observed difference between the regression calibration corrected and simulation-extrapolation corrected analysis in our motivating examples above, a simulation study was conducted to study the relative performance of regression calibration and simulation-extrapolation in a linear regression model and a logistic regression model. The relative performance was studied in terms of bias, mean squared error, and confidence interval coverage of the true effect. Subsection ‘Methods’ provides a general description and motivation of the scenarios studied, and an explanation of the specific parameters set in our simulation study. Subsection ‘Results’ presents the results of our simulation study.

### Methods

*Linear regression.* The relative performance of regression calibration and simulation-extrapolation for linear regression was studied before by Perrier et al. [5] and Batistatou et al. [27]. We aimed to extend these two former simulation studies by investigating the relative performance in scenarios other than those studied before. Perrier et al. and Batistatou et al. assumed relatively large sample sizes (i.e., 3000 and 1000, respectively), only four different values for the reliability of the error-prone exposure (i.e., 0.2 and 0.6 in the study by Perrier et al. and 0.2, 0.5 and 0.8 in the study by Batistatou et al.) and a small coefficient of determination for the exposure-outcome model (i.e., 0.004 and 0.0625, respectively). In addition, Perrier et al. studied the effect of increasing the number of replicate measures on the performance of regression calibration and simulation-extrapolation. However, since the replicate measures were pooled in the former study, an increase in the number of replicate measures led to a decrease in the measurement error bias. Moreover, Perrier et al. and Batistatou et al. only examined models with a single independent variable. Therefore, our simulation study focused on multivariable models, small sample sizes (i.e., smaller or equal to 1000) and relatively large reliability of the error-prone measurement (i.e., greater or equal to 0.625). In addition, the effect of a change in the coefficient of determination of the outcome model was tested. Furthermore, increasing the number of replicate measurements available was studied, without having the advantage of pooling the replicate measurements in our analysis.

*Data generating mechanism linear regression.* Inspired by our example of blood pressure and kidney function in pregnant women described in section ‘Review and motivating example’, we assumed the following data generating mechanisms for age, blood pressure (BP), error-prone blood pressure (BP^⁎^) and creatinine:

$$\mathrm{Age}\sim N\left(3225\right),\mathrm{BP}|\mathrm{Age}\sim N\left(120+\gamma \mathrm{Age},50\right),BP^{*}|\mathrm{BP}\sim N\left(\mathrm{BP},\tau^{2}\right)$$

and

$$\text{Creatinine}|\mathrm{BP},\mathrm{Age}\sim N\left(30+0.2\mathrm{BP}+0.2\mathrm{Age},\sigma^{2}\right)$$

The above defined data generating mechanism define that the error-prone blood pressure (BP^⁎^) has random measurement error with measurement error variance equal to *τ*^2^. In our simulation study, a `base scenario’ was assumed and in the consecutive scenarios studied, we changed one of the three parameters in the data generating mechanisms (i.e., *γ*, *τ*^2^ or *σ*^2^), the number of observations (i.e., n), or the number of replicate measures (i.e., k) (see Table 2). For each scenario, 5000 datasets were generated. The parameters settings of the base scenario were inspired by our example of blood pressure and kidney function in pregnant women [31]. In the base scenario, *n* = 500, *γ* = 0, *τ*^2^ = 30 and *σ*^2^= 100 (Table 2). Furthermore, we assumed that three replicate measures of the error-prone blood pressure measure were obtained in all individuals. From the parameter settings in the base scenario, it follows that the reliability of the error-prone measure is 0.625. Further, in the base scenario, the R-squared of the outcome model is 0.03, and the attenuation due to measurement error of the effect of the error-prone blood pressure on creatinine (given age) is equal to the reliability, i.e., 0.625.

**Table 2:** Simulation study settings linear regression.

Scenarios	Parameters of data generating mechanism^a^				
	*τ*^2^	*n*	*k*	*σ*^2^	*γ*
Base	30	500	3	100	0
Reliability^b^	200, 100, 50, 25, 20, 15, 10, 5	500	3	100	0
Sample size	30	125, 250, 1000, 10,000	3	100	0
Number of replicates	30	500	2, 5, 10	100	0
R-squared^c^	30	500	3	20, 5, 1	0
Covariate dependency^d^	30	500	3	100	1, 4, 8

a *τ*^2^: measurement error variance of the error-prone blood pressure measurement; *n*: number of observations in the main study; *k*: number of replicate error-prone measurements; *σ*^2^: residual variance of the outcome model; *γ*: association between blood pressure and age. The attenuation in the effect of blood pressure on creatinine due to random measurement error is equal to 50/(50 + *τ*^2^).

b Reliability is equal to (25*γ*^2^ + 50)/(25 *γ*^2^ + 50 + *τ*^2^).

c R-squared is equal to 1 − *σ*^2^/(0.4 × 50 + 10 + *σ*^2^).

d The effect of blood pressure on creatinine when age is not included in the model (crude model) is equal to 0.2 + 5*γ*/(25 *γ*^2^ + 50).

In each generated data set, the uncorrected effect was estimated using the first replicate measurement only. Subsequently, the corrected effect was estimated by application of regression calibration and simulation-extrapolation using the *R* package *mecor* [22] and *simex* [24], respectively. The measurement error variance was estimated using the replicate measures. Ninety-five percent CI's of the corrected effects were constructed using bootstrap resampling. Performance of the three different analyses were evaluated in terms of bias, mean squared error (MSE), and the proportion of 95% CIs that contained the true value of the estimand (coverage). Monte Carlo standard errors (MCSE) were calculated for all performance measures [33], using the *R* package *rsimsum* [34]. All code used for the simulation study is publicly available via www.github.com/LindaNab/simexvsmecor [35].

*Logistic regression.* The relative performance of regression calibration and simulation-extrapolation for logistic regression was studied before by Fung et al. [28]. Fung et al. assumed a relatively small sample size (i.e., 500) and relatively high reliability (i.e., 0.6 and 0.7). In our simulation study, we focus on parameters identical to the parameters varied in linear regression: reliability, sample size, number of replicates, pseudo R-squared (Nagelkerke) and covariate dependency.

*Data generating mechanism logistic regression.* Inspired by our example of sodium intake and hypertension described in section ‘Review and motivating example’, we assume the following data generating mechanisms for age, sodium intake (Na), error-prone sodium intake (Na^⁎^) and hypertension:

$$\mathrm{Age}\sim U\left[1880\right],\mathrm{Na}|\mathrm{Age}\sim N\left(4+\gamma \mathrm{Age},1\right),{\mathrm{Na}}^{*}|\mathrm{Na}\sim N\left(\mathrm{Na},\tau^{2}\right)$$

and

$$\text{Hypertension}|\mathrm{Na},\mathrm{Age}\sim \mathrm{Bern}\left(1,\frac{1}{1+e^{-p}}\right)$$

where *p* = − 7 + 0.1Na + *ϕ*Age.

The above defined data generating mechanism defines that the error-prone sodium intake (Na^⁎^) has random measurement error with measurement error variance equal to *τ*^2^. In our simulation study, a `base scenario’ was assumed and in the consecutive scenarios studied, we changed one of the three parameters in the data generating mechanisms (i.e., *τ*^2^, *ϕ* or *γ*), the number of observations (i.e., n), or the number of replicate measures (i.e., k) (see Table 3). For each scenario, 5000 datasets were generated. The parameters settings of the base scenario were inspired by our example of sodium intake and hypertension in adults [32]. In the base scenario, sample size was 4000, *τ*^2^ = 2, *ϕ* = 0.1 and *γ* = 0 (Table 2). Furthermore, we assumed that two replicate measures of the error-prone sodium intake measure were obtained in all individuals. From the parameter settings in the base scenario, it follows that the reliability of the error-prone measure is 0.33. Further, in the base scenario, the Nagelkerke pseudo R-squared of the outcome model was 0.4, and the attenuation due to measurement error of the effect of the error-prone sodium intake measure on hypertension (given age) was approximately equal to the reliability, i.e., 0.33.

**Table 3:** Simulation study settings logistic regression.

Scenarios	Parameters of data generating mechanism^a^				
	*τ*^2^	*n*	*k*	*ϕ*	*γ*
Base	2	4000	2	0.1	0
Reliability^b^	20, 10, 4, 1.5, 1, 0.5, 0.25, 0.1	4000	2	0.1	0
Sample size	2	500, 1000, 2000, 10,000	2	0.1	0
Number of replicates	2	4000	3, 5, 10	0.1	0
R-squared^c^	2	4000	2	0.06, 0.08, 0.2	0
Covariate dependency^d^	2	4000	2	0.1	0.01, 0.1, 0.2

a *τ*^2^: measurement error variance of the error-prone sodium intake measurement; *n*: number of observations in the main study; *k*: number of replicate error-prone measurements; *ϕ*: association between hypertension and age (given sodium intake); *γ*: association between sodium intake and age. The attenuation in the effect of sodium intake on hypertension due to random measurement error is equal to 1/(1 + *τ*^2^).

b Reliability is equal to $\left(\frac{1}{12}\gamma^{2}\;{\left(80-18\right)}^{2}+1\right)/\left(\frac{1}{12}\gamma^{2}\;{\left(80-18\right)}^{2}+1+\tau^{2}\right)$.

c Computational calculations show Nagelkerke R-squared is equal to 0.1, 0.3 and 0.7 for *ϕ* equal to 0.06, 0.08 and 0.2, respectively. In the base scenario, Nagelkerke R-squared is equal to 0.4.

d Computational calculations show that the effect of sodium intake on hypertension when age is not included in the model (crude model) is equal to 0.3, 0.8 and 0.6 for *γ* equal to 0.01, 0.1 and 0.2, respectively. In the base scenario, the effect of sodium intake on hypertension in the crude model is 0.06. Changing *γ* affects Nagelkerke R-squared, for *γ* equal to 0.1 and 0.2, Nagelkerke R-squared is 0.5. For *γ* equal to 0.01, Nagelkerke R-squared is comparable to the base scenario (0.4).

In each generated data set, the uncorrected effect was estimated using the first replicate measurement only. Subsequently, the corrected effect was estimated by application of regression calibration and simulation-extrapolation using regression calibration for logistic regression as described by Rosner et al. [23] and by use of the *R* package *simex* [24], respectively. The measurement error variance was estimated using the replicate measures. Ninety-five percent CI's of the corrected effects were constructed using bootstrap resampling. Performance of the three different analyses were evaluated in terms of bias, mean squared error (MSE), and the proportion of 95% CIs that contained the true value of the estimand (coverage). Monte Carlo standard errors (MCSE) were calculated for all performance measures [33], using the *R* package *rsimsum* [34]. All code used for the simulation study is publicly available via www.github.com/LindaNab/simexvsmecor [35].

### Results

**Fig. 1:**
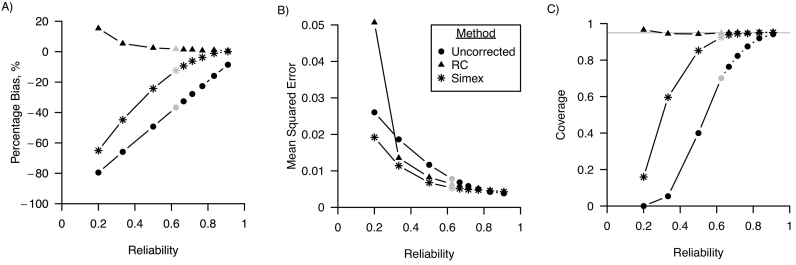
Performance in a linear regression model of regression calibration (RC), simulation-extrapolation (simex) and the analysis ignoring random measurement error for varying values of the reliability of the error-prone measure in terms of A) percentage bias; B) mean squared error and C) coverage. For all three performance measures, Monte Carlo standard errors were smaller than 0.01 in all scenarios. The grey points indicate the base scenario where reliability is assumed 0.625.

*Linear regression.*Fig. 1 shows the percentage bias, MSE and confidence interval coverage for varying values of the reliability of the error-prone measure. The uncorrected analysis was biased for all values of the reliability, and the percentage bias decreased when reliability increased. Regression calibration provided unbiased results when reliability was greater or equal to 0.33. Simulation-extrapolation provided biased results when reliability was smaller than 0.8. MSE was lower for simulation-extrapolation than for the uncorrected and regression calibration corrected analysis when reliability was equal to 0.2, and similar to MSE of regression calibration otherwise. Coverage of the 95% confidence intervals was at the nominal level for the regression calibration corrected analysis, and for the simulation-extrapolation corrected analysis when reliability was greater than or equal to 0.625.

**Fig. 2:**
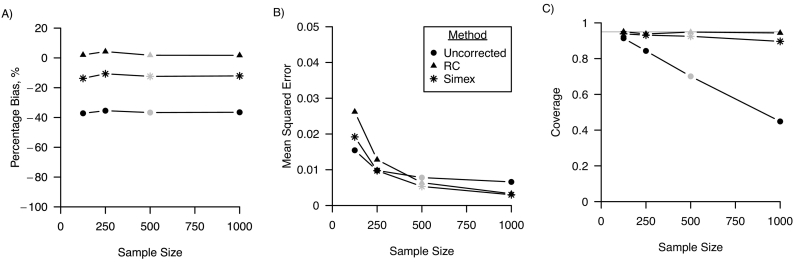
Performance of regression calibration (RC), simulation-extrapolation (simex) and the analysis ignoring random measurement error for varying sample sizes of the error-prone measure in terms of A) percentage bias; B) mean squared error and C) coverage. For all three performance measures, Monte Carlo standard errors were smaller than 0.01 in all scenarios. The grey points indicate the base scenario where sample size is assumed 500.

Fig. 2 shows the percentage bias, MSE and confidence interval coverage for varying samples sizes of the main study. A sample size of 125, and 250 only increased percentage bias minimally compared to the base scenario were sample size was 500. MSE was greater for smaller sample sizes, and MSE of the uncorrected analysis with a sample size of 125 was smaller than the regression calibration and simulation-extrapolation corrected analysis (0.015 vs 0.026 and 0.019, respectively, MCSE <0.005). Coverage was equal to the nominal level of 95% for regression calibration for all sample sizes, and the uncorrected analysis showed coverage levels that were subnominal, ranging between 45% and 91% (MCSE <0.01). Coverage of the 95% confidence intervals of the simulation-extrapolation corrected analysis was close to the nominal level of 95% except when sample size was 1000, in which case coverage was 90% (MCSE 0.004). A decline in confidence interval coverage for the simulation-extrapolation corrected analysis for larger sample sizes was confirmed by the scenario where sample size was 10,000, in which case coverage was 53% (MCSE 0.007) (not shown in the plots in Fig. 2).

**Fig. 3:**
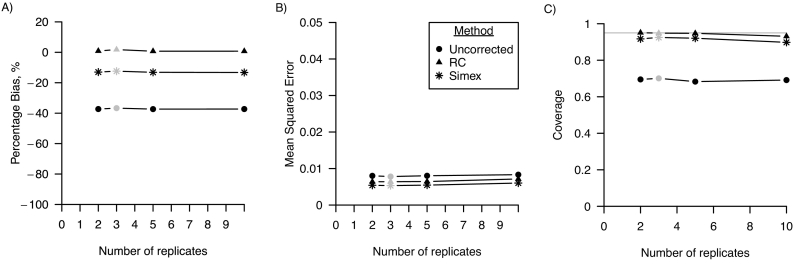
Performance in a linear regression model of regression calibration (RC), simulation-extrapolation (simex) and the analysis ignoring random measurement error for varying number of replicates of the error-prone measure in terms of A) percentage bias; B) mean squared error and C) coverage. For all three performance measures, Monte Carlo standard errors were smaller than 0.01 in all scenarios. The grey points indicate the base scenario where the number of replicates is assumed 3.

Fig. 3 shows that the number of replicates had no effect on the percentage bias, MSE and confidence interval coverage for varying number of replicates of the error-prone measure.

**Fig. 4:**
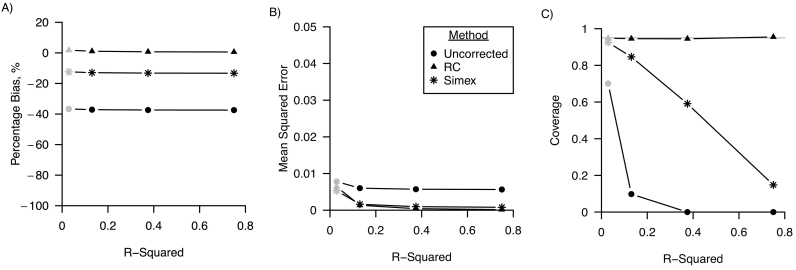
Performance in a linear regression model of regression calibration (RC), simulation-extrapolation (simex) and the analysis ignoring random measurement error for varying R-squared of the outcome model A) percentage bias; B) mean squared error and C) coverage. For all three performance measures, Monte Carlo standard errors were smaller than 0.01 in all scenarios. The grey points indicate the base scenario where R-squared is assumed 0.03.

Fig. 4 shows that R-squared had no effect on percentage bias, and only a minor decrease in MSE was found for increasing levels of R-squared. In addition, Fig. 4 shows that 95% confidence interval coverage was around the nominal level for the regression calibration corrected analysis for all values of the R-squared. However, for the uncorrected and the simulation-extrapolation corrected analysis, confidence interval coverage decreased for increasing values of R-squared. For R-squared equal to 0.75, confidence interval coverage decreased to 15% and 0% (MCSE <0.01) for the simulation-extrapolation corrected and the uncorrected analysis, respectively.

In the scenarios where a dependency between the covariate age and the exposure error-free blood pressure was introduced by changing parameter $\gamma$ in the data generating mechanism, the reliability of the error-prone measure was respectively 0.71, 0.94 and 0.98. However, percentage bias, MSE and confidence interval coverage of the uncorrected and corrected analyses were equal to the base scenario (the values in the base scenario are shown in e.g. Fig. 1). By introducing an effect of age on blood pressure, the total variance of the error-free blood pressure increased. Consequently, the extra variability in the error-prone blood pressure measurement due to measurement error was relatively smaller than in the base scenario. Hence, it seemed as if the error-prone variable was more reliable, though the attenuation due to random measurement error stayed constant at a rate of 0.625.

**Fig. 5:**
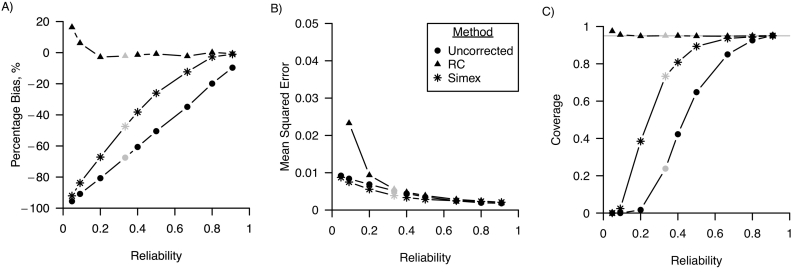
Performance in a logistic regression model of regression calibration (RC), simulation-extrapolation (simex) and the analysis ignoring random measurement error for varying values of the reliability of the error-prone measure in terms of A) percentage bias; B) mean squared error and C) coverage. For all three performance measures, Monte Carlo standard errors were smaller than 0.02 in all scenarios. The grey points indicate the base scenario where reliability is assumed 0.33. Mean squared error for RC and a reliability of 0.05 not shown (1.28, Monte Carlo standard error: 0.42).

*Logistic regression.*Fig. 5 shows the percentage bias, MSE and confidence interval coverage for varying values of the reliability of the error-prone measure. The uncorrected analysis was biased for all values of the reliability, and the percentage bias decreased when reliability increased. Regression calibration provided percentage bias close to null when reliability was greater or equal to 0.2. Simulation-extrapolation provided biased results when reliability was smaller than 0.8 and bias was close to null otherwise. MSE was similar for simulation-extrapolation and the uncorrected analysis across the range of reliability. MSE was greater for regression calibration than for the uncorrected and simulation-extrapolation analysis when reliability was equal to or smaller than 0.2, and similar otherwise. Coverage of the 95% confidence intervals was at the nominal level for the regression calibration corrected analysis across the range of reliability, and for the simulation-extrapolation corrected analysis when reliability was greater than or equal to 0.66.

**Fig. 6:**
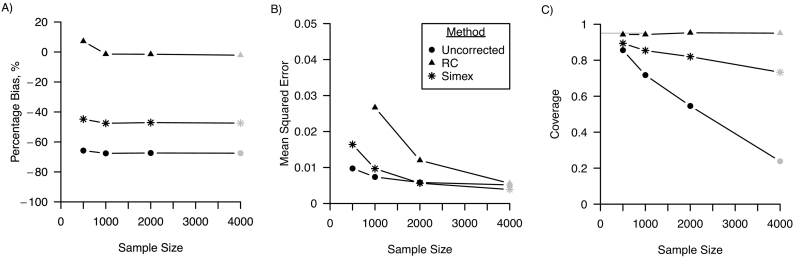
Performance in a logistic regression model of regression calibration (RC), simulation-extrapolation (simex) and the analysis ignoring random measurement error for varying sample sizes of the error-prone measure in terms of A) percentage bias; B) mean squared error and C) coverage. For all three performance measures, Monte Carlo standard errors were smaller than 0.01 in all scenarios. The grey points indicate the base scenario where sample size is assumed 4000. Mean squared error for RC and a sample size of 500 not shown (0.06, Monte Carlo standard error: <0.01).

Fig. 6 shows the percentage bias, MSE and confidence interval coverage for varying samples sizes of the main study. Percentage bias was increased for regression calibration for a sample size of 500 compared to the base scenario where sample size was 4000 (7% vs −2%, MCSE <0.01) and was similar otherwise. Percentage bias remained at a high level for simulation-extrapolation and the uncorrected analysis, ranging between −48% and − 45% (MCSE <0.01) and − 68% and − 66% (MCSE <0.01), respectively. MSE was greater for smaller sample sizes. For a sample size of 4000, MSE of regression calibration (ranging between 0.06 and 0.01, MCSE <0.01) was greater than for simulation-extrapolation and the uncorrected analysis, ranging between 0.01 and 0.02, MCSE <0.01 and 0.01, MCSE <0.01, respectively. Coverage was equal to the nominal level of 95% for regression calibration for all sample sizes. The uncorrected analysis and simulation-extrapolation were undercovered with coverage levels decreasing for increasing size of the sample size, ranging between 24% and 86% (MCSE <0.01) and 73% and 89% (MCSE <0.01), respectively. A decline in confidence interval coverage for the simulation-extrapolation corrected analysis for larger sample sizes was confirmed by the scenario where sample size was 10,000, in which case coverage was 50% (MCSE 0.02) (not shown in the plots in Fig. 6).

**Fig. 7:**
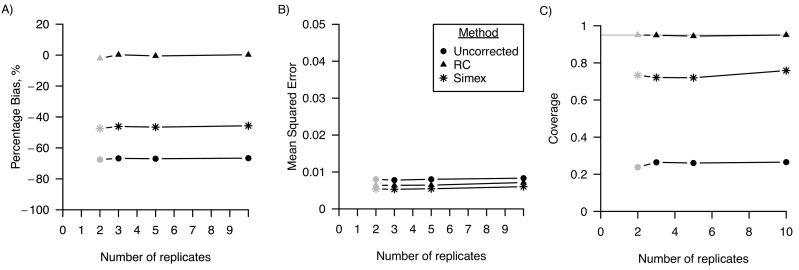
Performance in a logistic regression model of regression calibration (RC), simulation-extrapolation (simex) and the analysis ignoring random measurement error for varying number of replicates of the error-prone measure in terms of A) percentage bias; B) mean squared error and C) coverage. For all three performance measures, Monte Carlo standard errors were smaller than 0.01 in all scenarios. The grey points indicate the base scenario where the number of replicates is assumed 2.

Fig. 7 shows that the number of replicates had no effect on the percentage bias, MSE and confidence interval coverage for varying number of replicates of the error-prone measure.

The effect of changing R-squared in a logistic regression model had a less pronounced effect on percentage bias, MSE and confidence interval coverage, than in linear regression. Details can be found in the Appendix A.

In the scenarios where a dependency between the covariate age and the error-free exposure sodium intake was introduced by changing parameter *γ* in the data generating mechanism, the reliability of the error-prone measure was respectively 0.34, 0.68, 0.87. However, similar to what was seen for linear regression, percentage bias, MSE and confidence interval coverage of the uncorrected and corrected analyses were equal to the base scenario (the values in the base scenario are shown in e.g. Fig. 5).

## Sensitivity analysis in the absence of validation data

In the first example introduced in section ‘Review and motivating example’, replicate measurements of the error-prone systolic blood pressure were available. Nevertheless, validation data in the form of replicate measurements may not always be available. When random measurement error in a covariate is suspected in the absence of such validation data, a sensitivity analysis could be conducted using regression calibration or simulation-extrapolation. A general framework for conducting sensitivity analysis for random measurement error is described here, where we assume that the input of the sensitivity analysis, i.e., the measurement error variance and its uncertainty, are obtained from literature or expert knowledge. Next, a distribution for the measurement error variance is assumed, e.g., a uniform, triangular, or trapezidiol distribution [29]. Subsequently, regression calibration or simulation-extrapolation are applied to the data for measurement error correction, informed by the measurement error variance and its distribution. Finally, the results of the application of measurement error correction are presented, and conclusions drawn about the sensitivity of the results to measurement error.

### Sensitivity analysis for measurement error in the example of blood pressure and kidney function in pregnant women

Suppose that in the example of the relation between blood pressure and kidney function in pregnant women discussed in section ‘Review and motivating example’ (example 1), only the first systolic blood pressure measurement was available. A 10 mmHg increase in systolic blood pressure was associated with a 1.18 μmol/L (95% CI 0.14;2.23) increase in serum creatinine. Random measurement error, however, could have been suspected in the single systolic blood pressure measurement and suppose the sensitivity of the results to the measurement error was studied. Suppose it was assumed that the variance of the measurement error in systolic blood pressure was equal to 48 mmHg, with a minimum of 37 mmHg and a maximum of 59 mmHg. Additionally, suppose a triangular distribution was assumed for the measurement error variance, meaning that most weight was put on 48 mmHg, and the weight was gradually reduced until it reached the assumed minimum and maximum level. The triangular distribution was sampled in accordance with Lash et al. [29].

**Fig. 8:**
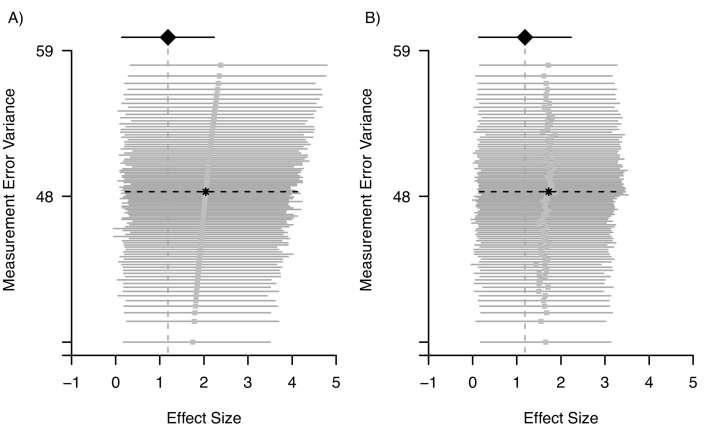
Sensitivity analysis for the association between blood pressure and kidney function in pregnant women [dataset] [31] (example 1) by application of regression calibration (panel A) and simulation-extrapolation (panel B). The uncorrected association and 95% confidence interval are depicted with a diamond and a solid black line, the measurement error corrected associations and 95% confidence intervals are depicted with a square and a solid grey line. The distribution of the measurement error variance is triangular. For reference, the measurement error corrected association and 95% confidence interval using the replicates data is depicted with a star and a dashed black line.

Fig. 8 shows the results of the application of regression calibration and simulation-extrapolation informed by the triangular distribution. For regression calibration, a clear pattern was obtained. The corrected effect estimates increased for larger values of the measurement error variance, with the effect estimates ranging from 1.75–2.38, with a median of 2.03. In addition, the associated lower limits of the confidence intervals consistently suggest an association between blood pressure and creatinine. In comparison, simulation-extrapolation did not show a clear pattern in the corrected effect estimates. The corrected effect estimates ranged from 1.43–1.88, with a median of 1.70. Fig. 8 shows that the sampling variability that is inherent to simulation-extrapolation causes more variability in the effect estimates compared to the variability due to random measurement error. Nevertheless, the lower limits of the associated confidence intervals again consistently suggest an association between blood pressure and creatinine levels.

In the sensitivity analysis described here, results were presented graphically, and no summary estimate was shown. In the presented plots, the variability around the corrected estimates was shown graphically. Alternatively, one can incorporate the variability around the corrected estimates in a so-called probabilistic bias analysis, by repeatedly sampling the corrected estimate from a distribution. Usually, it is assumed that the corrected estimate is normally distributed with mean equal to the point estimate and standard deviation equal to the standard error of the estimate. The sampled values can be presented by plotting the distribution of corrected estimates. There is a close resemblance between a probabilistic bias analysis and a Bayesian bias analysis with an uninformative prior for the association under study. We refer to MacLehose et al. for a thorough discussion of this issue [36]. More information about probabilistic bias analysis can be found in e.g., the book by Lash et al. [29] and more details about a Bayesian analysis for measurement error can be found in e.g., the book by Gustafson et al. [16].

## Discussion

This paper compared regression calibration and simulation-extrapolation for sensitivity analysis for random measurement error in an exposure variable. A simulation study showed that with correct assumptions about the measurement error variance, regression calibration was generally unbiased for linear and logistic regression when the reliability of the error-prone measurement was greater than 0.2. The bias in the regression calibration corrected analysis for linear regression for low reliability was unexpected but may be explained by the instability of regression calibration when the correction factor is close to null. The bias in the regression calibration corrected analysis for logistic regression for low reliability was expected as Kuha showed that regression calibration for logistic regression is only approximately unbiased when the effect of the exposure on the outcome is ‘small to moderate’ and/or the measurement error variance ‘small’ [30]. Moreover, the uncorrected and simulation-extrapolation corrected analysis were generally biased, with higher bias for lower reliability of the error-prone exposure. Confidence interval coverage for regression calibration was generally close to the nominal level of 95% for linear and logistic regression. On the contrary, the confidence interval coverage of the simulation-extrapolation corrected and uncorrected analysis were subnominal.

The uncorrected analysis was shown more efficient than the corrected analyses for linear regression in settings where reliability was low (i.e., 0.2) or sample size small (i.e., 125). This observation is the result of the substantially smaller variance of the uncorrected analysis compared to the corrected analyses, which outweighs the larger bias for the uncorrected analysis. This is sometimes referred as the bias versus variance tradeoff, we refer to chapter 3 of the book by Carroll et al. for a detailed discussion [13]. The same pattern was obtained for logistic regression. In addition, simulation-extrapolation showed a small gain in efficiency over regression calibration for linear regression in settings where reliability was low (i.e., 0.2) or sample size small (i.e., 250 and 125), and similar efficiency otherwise. The efficiency gain of simulation-extrapolation over regression calibration obtained for linear regression was not seen in logistic regression because of the large bias in the simulation-extrapolation analysis in most settings.

The results of our simulation study were in line with the results of three previous simulation studies: the corrected analyses showed lower percentages bias compared to the uncorrected analysis and the simulation-extrapolation corrected analysis showed higher percentage bias compared to regression calibration [5,27,28]. However, important differences were observed. First, simulation-extrapolation showed a small gain in efficiency over regression calibration for linear regression in some settings, which was not found in the previous simulation studies. The sample sizes for which this gain in efficiency for simulation-extrapolation was observed (i.e., 125, 250 and 500) were smaller than those assumed by Perrier et al. [5] and Batistatou et al. [27] (i.e., 3000 and 1000, respectively), which may explain the found difference. Second, our simulation showed no effect of the number of replicates on bias. While the simulation study by Perrier et al. showed that an increasing number of replicates reduced bias in the corrected analyses [5]. This difference is explained by the fact that in the study by Perrier et al., the replicate measures were pooled before applying measurement error correction. By pooling the replicate measures with random measurement error, the measurement error variance is reduced. Therefore, bias decreased in the study by Perrier et al. with the availability of more replicate measures. This effect however is solely due to pooling the replicates measures and not due to a more precise estimate of the measurement error variance, as was shown by our results. Third, the simulation study by Fung et al. [28] showed that by increasing the correlation between the exposure and a covariate, the attenuation in the uncorrected analysis increased toward the null value. In comparison, our simulation study showed no effect of covariate dependency on bias in the uncorrected analysis. This is explained by the fact that in our simulation study, the variance of the exposure given the covariate was kept constant, while the total variance of the exposure was varied by introducing the covariate dependency (i.e., changing *γ* in the data generating mechanism). In comparison, in the simulation study by Fung et al., the variance of the exposure given the covariate was varied, resulting in an increase in the attenuation factor.

Our simulation study showed that percentage bias in the uncorrected analysis was equal to 1 minus the reliability of the error-prone measure times 100, in line with theory [8,9]. The reliability of an error-prone measure equals the variance of the error-free measure divided by the variance of the error-prone measure. For example, in Fig. 1, bias in the uncorrected analysis was equal to 80% for a reliability equal to 0.2. The uncorrected effect estimate is equal to 0.2 times the estimand 0.2, i.e., 0.04. From that, it follows that the bias is equal to 0.2–0.04 = 0.16, which is 80% of 0.2. It is, however, important to note that the percentage bias is not equal to 1 minus the reliability of the error-prone measure when the total variance of the error-free measure depends on a covariate that is also included in the outcome model. For example, in our simulation study, the association between creatinine and systolic blood pressure given age was estimated. When a dependency between systolic blood pressure and age was introduced, the reliability increased to a maximum of 0.98 while the percentage bias in the uncorrected analysis was constant at 62.5%. A formula for the attenuation in the effect estimate due to random measurement error in multivariable models can be found in e.g. [13].

In our simulation study, the measurement error variance used to correct for the random measurement error was estimated using replicate measures. However, we assumed that these replicate measures were solely available to estimate the measurement error variance, to mimic a setting where such validation data are not available, yet unbiased information is available about the measurement error variance. In future studies, this work could be extended to settings where the measurement error is estimated with bias, and to settings where the measurement error model is misspecified. Also non-random measurement error, e.g., systematic measurement error and differential measurement error, which was not the topic of this study, could be considered in future work. Simulation-extrapolation is not suited for the correction of measurement other than random measurement error, and for regression calibration, the full calibration model needs to be specified. We refer to Nab et al. [22] for a specification of the calibration model in case of systematic measurement error and what validation data can be used to estimate the calibration model. In addition, in our simulation study, models with one covariate and normal distributed measurement error were considered. The results of our study can be extended to settings with more covariates and measurement error with a skewed or heavy-tailed distribution. The covariate in our data generating mechanism can be viewed as a summary of a larger set of variables. What is more, transformations can turn a skewed or heavy-tailed measurement error distribution into a distribution that is closer to the normal distribution, as proposed by Carrol et al. [13]. Alternatively, adopted versions of regression calibration for heteroscedastic measurement error could be used [37].

Our study discussed measurement error correction methods for sensitivity analysis of random measurement error in a continuous exposure. For a categorical exposure, measurement error will lead to misclassification of the exposure. In this setting, different measurement error correction methods could be used. For example, the misclassification simulation-extrapolation [38], available in the *R* package *simex* [24]. Or alternatively, the probabilistic sensitivity analysis of misclassified binary variables described by Fox et al. [39].

Our study explored the performance of regression calibration and simulation-extrapolation for the correction of random measurement error in a linear regression model and a logistic regression model. For a survival model, the effects of random measurement error cannot be derived exactly as shown in chapter 14 of the book by Carroll et al. [13]. In particular, regression calibration gives approximately consistent estimates only in cases of a rare outcome, and for a hazard ratio of ‘small to moderate’ size or ‘small’ measurement error variance [40]. Xie et al. proposed a more flexible regression calibration approach for Cox regression that is referred to as ‘risk set regression calibration’ [41]. Alterations of the simulation-extrapolation method have been proposed for proportional hazard models [42] and accelerated failure time models [43]. For Poisson regression, regression calibration only provides estimates that are approximately unbiased, and usually works well, when the effect of the exposure on the outcome is ‘small to moderate’ or the measurement error variance ‘small’ [13]. Fung et al. compare regression calibration and simulation-extrapolation for Poisson regression and concluded that regression calibration performed best in all scenarios considered [44].

The Achilles heel of simulation-extrapolation is the extrapolation step [3]. Our simulation study uses a quadratic extrapolation. Lockwood et al. demonstrate the use of a quartic extrapolation, that may reduce bias in the simulation-extrapolation estimator [45].

In the example presented in section 4, the five steps of a sensitivity analysis for random exposure measurement error were described: 1) quantify the measurement error variance and its uncertainty; 2) specify the distribution of the measurement error variance; 3) perform measurement error correction by means of regression calibration or simulation-extrapolation; 4) visualise the results, and 5) draw conclusions. A sensitivity analysis using regression calibration showed that the higher the measurement error variance, the more the corrected effect estimate departs from the null, which is in line with the literature [8,9]. In the sensitivity analysis using simulation-extrapolation, the variability in the corrected effect estimates due to the sampling variability inherent to simulation-extrapolation exceeded the variability in the corrected effect estimates due to the assumed measurement error variance. Our simulation results showed that the regression calibration estimator is generally unbiased while the simulation-extrapolation estimator is. In contrast, simulation-extrapolation showed a small efficiency gain over regression calibration. Despite the efficiency gain for simulation-extrapolation, we recommend the use of regression calibration for sensitivity analysis. In a sensitivity analysis, focus is on the quantification of the impact of measurement error on the point estimate, and the confidence interval width may be of lesser importance.

In conclusion, regression calibration and simulation-extrapolation are suited for sensitivity analysis for random measurement error. It is difficult to say anything definite about the behavior of regression calibration and simulation-extrapolation based on a handful of simulation studies. We have, however, covered many aspects in our simulation study, i.e., reliability, sample size, number of replicates, explained variance of the outcome model and covariate dependency. The pattern is so pronounced and in accordance with findings of former simulation studies [5,27,28], that we think it is safe to say that regression calibration may be preferred over simulation-extrapolation. Nevertheless, if researchers want to compare simulation-extrapolation with regression calibration in simulation settings that are closer to their intended field of application, then we provided our simulation code, which can be modified easily to allow for investigation of such scenarios.

## Data and code availability

The data and code used for the simulation study have been made publicly available and can be accessed via www.github.com/LindaNab/simexvsmecor.

## Source of funding

This study was supported by grants from the Netherlands Organization for Scientific Research (ZonMW-Vidi project 917.16.430) and Leiden University Medical Center.

## Declaration of Competing Interest

The authors declare no conflicts of interest.

## Appendix A Supplementary data

Supplementary material Image 1
