# Supplementary material for: Sensitivity analysis for random measurement error using regression calibration and simulation-extrapolation
Source: Glob Epidemiol. 2021 Nov 21;3:100067. doi: 10.1016/j.gloepi.2021.100067 (PMC10446124; doi:10.1016/j.gloepi.2021.100067)
Supplement: Supplementary file 1 — Supplementary material [file mmc1.pdf]

A1 Additional simulation study results

This appendix shows the additional results of the simulation study from the paper 'Sensitivity analysis for random measurement error using regression calibration and simulation-extrapolation' by Linda Nab et al. In specific, here, we show the results of the relative performance of the uncorrected, regression calibration and simulation-extrapolation corrected analysis, for a logistic regression for varying values of the Pseudo R-squared of the outcome model. Pseudo R-squared is varied by varying the parameter ϕ in the data generating mechanism for logistic regression discussed in subsection 'methods' of the section 'simulation study' of the main paper. Figure A1 shows that bias remains stable compared to the base scenario for varying values of Pseudo R-squared, except for Pseudo R-squared equal to 0.12. Mean squared error was higher for regression calibration than for the uncorrected or simulation-extrapolation corrected analysis for Pseudo R-squared equal to 0.12, 0.25 and 0.69. Coverage remained at the nominal level of 95% for regression calibration, and was subnominal for simulation-extrapolation and the uncorrected analysis with values ranging between 73%-85% and 24%-68%, respectively.

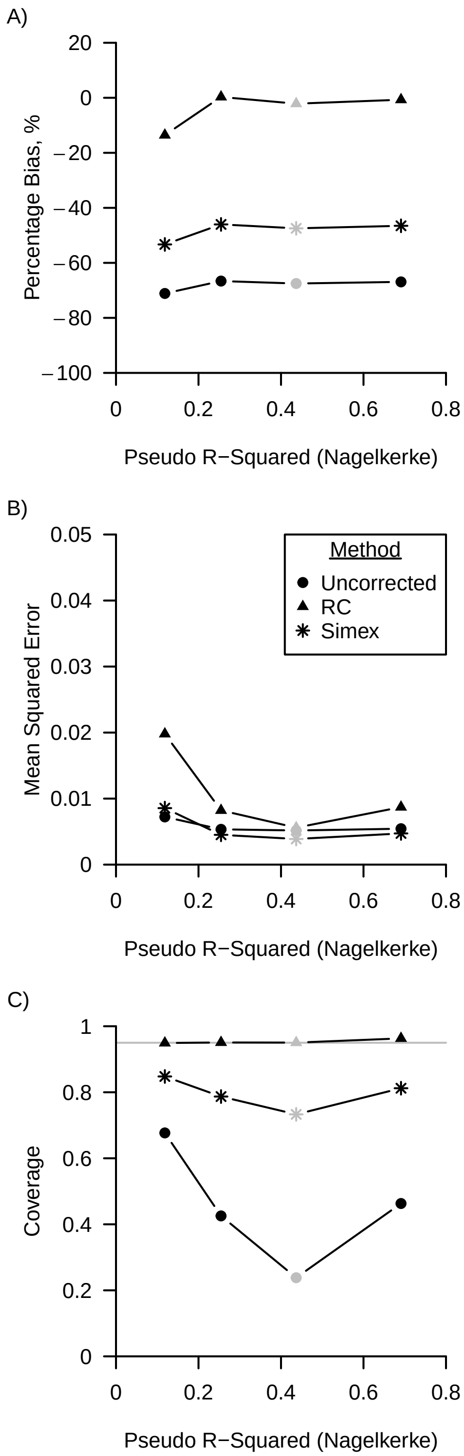

Figure A1. Performance in a logistic regression model of regression calibration (RC), simulation-extrapolation (simex) and the analysis ignoring random measurement error for varying R-squared (Nagelkerke) of the outcome model A) percentage bias; B) mean squared error and C) coverage. For all three performance measures, Monte Carlo standard errors were smaller than 0.01 in all scenarios. The grey points indicate the base scenario where R-squared is assumed 0.42.
